# Dr. Thomas H. Hallett

**Published:** 1917-05

**Authors:** 


**Dr. Thomas H. Hallett,** Buffalo 1890, died at his home in Clyde, N. Y., Feb. 11, of pernicious anaemia, aged 56.

